# gcFront: a tool for determining a Pareto front of growth-coupled cell factory designs

**DOI:** 10.1093/bioinformatics/btac376

**Published:** 2022-06-01

**Authors:** Laurence Legon, Christophe Corre, Declan G Bates, Ahmad A Mannan

**Affiliations:** Warwick Integrative Synthetic Biology Centre, School of Engineering, University of Warwick, Coventry CV4 7AL, UK; Warwick Integrative Synthetic Biology Centre, School of Life Sciences, University of Warwick, Coventry CV4 7AL, UK; Warwick Integrative Synthetic Biology Centre, School of Life Sciences, University of Warwick, Coventry CV4 7AL, UK; Warwick Integrative Synthetic Biology Centre, School of Engineering, University of Warwick, Coventry CV4 7AL, UK; Warwick Integrative Synthetic Biology Centre, School of Engineering, University of Warwick, Coventry CV4 7AL, UK

## Abstract

**Motivation:**

A widely applicable strategy to create cell factories is to knockout (KO) genes or reactions to redirect cell metabolism so that chemical synthesis is made obligatory when the cell grows at its maximum rate. Synthesis is thus growth-coupled, and the stronger the coupling the more deleterious any impediments in synthesis are to cell growth, making high producer phenotypes evolutionarily robust. Additionally, we desire that these strains grow and synthesize at high rates. Genome-scale metabolic models can be used to explore and identify KOs that growth-couple synthesis, but these are rare in an immense design space, making the search difficult and slow.

**Results:**

To address this multi-objective optimization problem, we developed a software tool named gcFront—using a genetic algorithm it explores KOs that maximize cell growth, product synthesis and coupling strength. Moreover, our measure of coupling strength facilitates the search so that gcFront not only finds a growth-coupled design in minutes but also outputs many alternative Pareto optimal designs from a single run—granting users flexibility in selecting designs to take to the lab.

**Availability and implementation:**

gcFront, with documentation and a workable tutorial, is freely available at GitHub: https://github.com/lLegon/gcFront and archived at Zenodo, DOI: 10.5281/zenodo.5557755.

**Supplementary information:**

Supplementary data are available at *Bioinformatics* online.

## 1 Introduction

Genome-scale constraint-based models (GSMs) are used to explore gene or reaction knockouts (KOs) that redirect cell metabolism to chemical overproduction (Maia *et al.,* 2016). A promising strategy for enabling robust production seeks KO combinations that couple chemical synthesis with cell growth so that it is made obligatory at maximum growth rate (Feist *et al.,* 2010). KOs can disrupt metabolism to result in poorer performance than predicted, but growth coupling enables the selection of higher producing phenotypes by selecting faster growing cells through adaptive laboratory evolution (ALE). KOs by gene deletion are easily implemented in the lab, and since they remain fixed in the face of evolution, as opposed to engineering changes in gene expression, ALE has been shown to find strains with synthesis and growth rates near the optimal values predicted from GSMs (Tokuyama *et al.,* 2018). However, if the coupling is weak, cells will not synthesize the product unless they grow close to their theoretical maximum. Instead, KOs that create a strong coupling result in evolutionarily robust phenotypes with robust synthesis, and so are particularly appealing. Specifically, stronger coupling will strongly impair growth for small impediments in product synthesis, so higher producers will be reselected over evolutionary time, and it also helps conserve synthesis rates even if cells grow at suboptimal rates, for instance in large fermenters (Supplementary Fig. S1). In addition to strong coupling, we also desire that these strains grow fast but also synthesize rapidly. Identifying the KO sets, i.e., designs, that maximize these criteria is a multi-objective optimization problem. However, there are inherent trade-offs between some of these objectives, so solving this problem will give a set of alternative optimal designs where for each design each objective cannot be improved without sacrificing some of the others. This is known as a Pareto front of optimal designs. Multi-objective optimization has been applied in metabolic engineering, for instance to kinetic models to find Pareto optimal reaction kinetics that maximize synthesis (Sendín *et al.,* 2006; Vera *et al.,* 2003), and tools have been developed for use on GSMs to determine genetic manipulations to maximize growth and synthesis (Andrade et al., 2020; Patané *et al.,* 2019). Other tools have been developed to find growth-coupled designs (Alter and Ebert, 2019; Feist *et al.,* 2010; Ohno *et al.,* 2014), yet there is no tool to determine optimal designs that maximize coupling strength, growth and synthesis, in order to create evolutionarily robust strains with high productivity and robust synthesis—critical for industrial application. Moreover, though growth coupling is a widely applicable strategy (von Kamp and Klamt, 2017) KOs enabling this are rare, making the search for them difficult and slow (Ohno *et al.,* 2014). To address this key gap and problem, we developed a user-friendly software tool named gcFront that uses a genetic algorithm to search for KOs that maximize these three objectives, for any chemical and host of interest. Moreover, our proposed measure of coupling strength facilitates the search through the design space, so a run of gcFront outputs many Pareto optimal designs in reasonable timeframes.

## 2 The gcFront workflow

gcFront works in MATLAB, with dependencies on the COBRA toolbox (Heirendt *et al.*, 2019) for analysis of a compatible GSM; and the MATLAB Global Optimization toolbox for solving the multi-objective optimization problem (Supplementary Note S1A). The workflow, detailed in Supplementary Note S1B and Figure S2, entails four key steps.

***Inputs***: Two interactive windows allow the user to define the GSM, target metabolite product or its exchange reaction and optional inputs (Supplementary Table S1), such as maximum number of KOs and search time.

***Pre-processing***: To reduce the search space of reactions, gcFront automatically identifies and removes dead reactions, lumps unbranched pathways into composite reactions and excludes in silico essential single KOs for growth or synthesis.

***Solving the optimization problem***: To determine growth-coupled designs, gcFront solves the multi-objective optimization problem defined in Supplementary Note S2A. Our measure of coupling strength shapes the search landscape; it defines weak and strong coupling but also distinguishes between uncoupled designs (Supplementary Note S2B and Fig. S3a). It assigns higher values to KOs that reduce the cost to growth for increases in the maximum allowable synthesis, thus driving a bias to gc-designs (Supplementary Fig. S3b and c) to ease the search.

***Post-processing and output***: On termination (conditions in Supplementary Table S1), many Pareto optimal KO sets are found from a single run. Some proposed designs may contain redundant KOs, so to minimize the number of KOs of each design any KO that can be removed from those designs without any loss in performance is removed. The Pareto front of all designs (KOs) and their performance is then output to an interactive plot, a table in the command window and a .csv file. Users can select designs they deem suitable for their chemical and host of interest, based on bespoke combinations of the performance metrics. A tutorial is given in Supplementary Note S3.

## 3 Comparative performance assessment

**Fig. 1.**
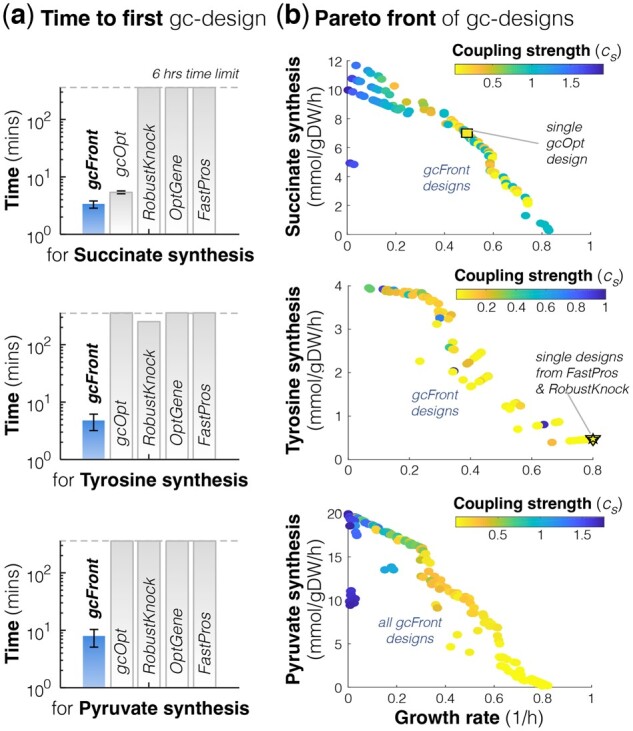
**gcFront finds many Pareto optimal growth-coupled designs, faster and with superior performance versus other algorithms.** The speed and designs found from 6-h runs of gcFront were compared to those of RobustKnock, gcOpt, FastPros and OptGene (see Supplementary Note S4) on a MacBook Pro (2.3 GHz Quad-Core Intel core i5 processor, 8 GB 2133 MHz LPDDR3 RAM). Designs were based on KOs of only non-essential, gene-associated reactions, for the synthesis of three example products: succinate, tyrosine and pyruvate from the *E.coli* iML1515 GSM model, in aerobic, minimal media with glucose. (**a**) Time to identify the first gc-design from each procedure. Due to the stochastic nature of searching using the genetic algorithm in OptGene and gcFront, the average (bars) and standard deviation (error bars) of times are reported from three runs (*N* = 3, ±SD). (**b**) Pareto fronts of all gc-designs found from three 6-h runs

To test gcFront’s performance, we compared it to other MATLAB-based procedures that identify growth-coupled (gc-)designs, including RobustKnock (Tepper and Shlomi, 2010) as implemented in OptPipe (Hartmann *et al.*, 2017); gcOpt (Alter and Ebert, 2019); FastPros (Ohno *et al.*, 2014) and OptGene (Patil *et al.*, 2005) as implemented in COBRA (Heirendt *et al.*, 2019). We ran each for 6 h and saved the gc-designs found and the time they needed to find their first gc-design, while repeating this three times for gcFront and OptGene because of the stochastic nature of searching with a genetic algorithm. For a fair comparison, we ran each algorithm using the *Escherichia* *coli* GSM model iML1515 (Monk *et al.,* 2017), for non-essential reaction KOs [in silico and based on Goodall *et al.* (2018)], for synthesis of succinate, tyrosine and pyruvate, as example products (detailed in Supplementary Notes S4 and Fig. S1). gcFront found the first gc-design in 38% less time than gcOpt for succinate synthesis, 98% less time than RobustKnock for tyrosine synthesis, and orders of magnitude less time than the other methods and products (Fig. 1a, Supplementary Data). Its power was especially apparent when searching for designs of tyrosine and pyruvate synthesis—still finding designs in minutes despite these designs, of at least six KOs, being rarer versus three KOs found for succinate (Supplementary Fig. S4). Furthermore, though the single gc-design found with other methods lay near the Pareto front of gc-designs from gcFront, gcFront offered many designs that achieved at least higher coupling strength (Fig. 1b, Supplementary Data).

## 4 Discussion

gcFront can find a multitude of Pareto optimal growth-coupled designs for evolutionarily robust cell factories, from a single run, in a computationally efficient manner. With the key input being the genome-scale metabolic network model of the cell host with the biochemistry of the engineered product synthesis pathway, gcFront should be widely applicable for designing growth-coupled synthesis of any compound, from any host, and so drive the design step in the design-build-test-learn cycle (Carbonell *et al.*, 2018). Since each design provides a different balance between the maximized objectives, the user has the flexibility to select designs with the balance they deem most suitable to the cell host and chemical product of interest, e.g. sacrifice growth for stronger coupling and synthesis, for instance for more robust pyruvate synthesis (Fig. 1b); or sacrifice synthesis for higher growth and stronger coupling, for instance for higher volumetric productivity with robust synthesis of succinate (Fig. 1b)—making it widely applicable to different contexts. gcFront is also user friendly, but versatile—the interactive user interface means no coding is required, making gcFront easy to use out-of-the-box, yet because it is a function in the MATLAB environment it can be easily integrated downstream of pathway designing tools, such as COBRA toolbox (Heirendt *et al.*, 2019) and RetroPath2.0 (Delépine *et al.*, 2018). Importantly, since gcFront proposes KOs for growth coupling and not changes in gene expression, strain construction and evolution is more easily automated. With recent technical advances in Synthetic Biology and lab robotics, subsequent to user-led design selection, we envision that gcFront can be integrated in pipelines upstream of robotics platforms for automated plasmid construction and transformation with a robot performing CRISPR-Cas9-based KOs (Suckling *et al.*, 2018), and automated ALE with liquid handling robotics, e.g. RoboLector (Radek *et al.*, 2017)—making gcFront a tool for the future of creating microbial cell factories.

## Author contributions

L.L., A.A.M.: developed theory; L.L.: developed code, ran procedures, plotted results; A.A.M., D.G.B.: designed and supervised the research; all authors: wrote the paper.

## Data availability

The data underlying this article are available in the article and its online Supplementary Information and Supplementary Data files.

## Funding

This work was supported by funds from the Engineering and Physical Sciences Research Council [EP/L016494/1] and Biotechnology and Biological Sciences Research Council [BB/M017982/1].

*Conflict of Interest*: none declared.

## Supplementary Material

btac376_Supplementary_Data

## References

[btac376-B1] Alter T.B. , Ebert B.E. (2019) Determination of growth-coupling strategies and their underlying principles. BMC Bioinformatics, 20, 447. 31462231 10.1186/s12859-019-2946-7 PMC6714386

[btac376-B2] Andrade R. et al (2020) MOMO—multi-objective metabolic mixed integer optimization: application to yeast strain engineering. BMC Bioinformatics, 21, 69. 32093622 10.1186/s12859-020-3377-1 PMC7041195

[btac376-B3] Carbonell P. et al (2018) An automated design-build-test-learn pipeline for enhanced microbial production of fine chemicals. Commun. Biol., 1, 66. 30271948 10.1038/s42003-018-0076-9 PMC6123781

[btac376-B4] Delépine B. et al (2018) RetroPath2.0: a retrosynthesis workflow for metabolic engineers. Metab. Eng., 45, 158–170. 29233745 10.1016/j.ymben.2017.12.002

[btac376-B5] Feist A.M. et al (2010) Model-driven evaluation of the production potential for growth-coupled products of *Escherichia coli*. Metab. Eng., 12, 173–186. 19840862 10.1016/j.ymben.2009.10.003 PMC3125152

[btac376-B6] Goodall E.C.A. et al (2018) The essential genome of *Escherichia coli* K-12. MBio, 9, e02096-17. 29463657 10.1128/mBio.02096-17 PMC5821084

[btac376-B7] Hartmann A. et al (2017) OptPipe—a pipeline for optimizing metabolic engineering targets. BMC Syst. Biol., 11, 143. 29268790 10.1186/s12918-017-0515-0 PMC5740890

[btac376-B8] Heirendt L. et al (2019) Creation and analysis of biochemical constraint-based models using the COBRA toolbox v.3.0. Nat. Protoc., 14, 639–702. 30787451 10.1038/s41596-018-0098-2 PMC6635304

[btac376-B9] Legon L. et al (2022) *lLegon/gcFront: gcFront V1.2 (V1.2_Publication_version)*. Zenodo. 10.5281/zenodo.5557755.

[btac376-B10] Maia P. et al (2016) In silico constraint-based strain optimization methods: the quest for optimal cell factories. Microbiol. Mol. Biol. Rev., 80, 45–67. 26609052 10.1128/MMBR.00014-15 PMC4711187

[btac376-B11] Monk J.M. et al (2017) A knowledgebase that computes *Escherichia coli* traits. Nat. Biotechnol., 35, 904–908. 29020004 10.1038/nbt.3956 PMC6521705

[btac376-B12] Ohno S. et al (2014) FastPros: screening of reaction knockout strategies for metabolic engineering. Bioinformatics, 30, 981–987. 24257186 10.1093/bioinformatics/btt672 PMC3967105

[btac376-B13] Patané A. et al (2019) Multi-objective optimization of genome-scale metabolic models: the case of ethanol production. Ann. Oper. Res., 276, 211–227.

[btac376-B14] Patil K.R. et al (2005) Evolutionary programming as a platform for in silico metabolic engineering. BMC Bioinformatics, 6, 308. 16375763 10.1186/1471-2105-6-308 PMC1327682

[btac376-B15] Radek A. et al (2017) Miniaturized and automated adaptive laboratory evolution: evolving *Corynebacterium glutamicum* towards an improved D-xylose utilization. Bioresour. Technol., 245, 1377–1385. 28552568 10.1016/j.biortech.2017.05.055

[btac376-B16] Sendín O.H. et al (2006) Model based optimization of biochemical systems using multiple objectives: a comparison of several solution strategies. Math. Comput. Model. Dyn. Syst., 12, 469–487.

[btac376-B17] Suckling L. et al (2018) Automated high-throughput transformation of bacteria cells. Appl. Note CyBio^®^ Felix, **1**, 1–7.

[btac376-B18] Tepper N. , Shlomi T. (2010) Predicting metabolic engineering knockout strategies for chemical production: accounting for competing pathways. Bioinformatics, 26, 536–543. 20031969 10.1093/bioinformatics/btp704

[btac376-B19] Tokuyama K. et al (2018) Application of adaptive laboratory evolution to overcome a flux limitation in an *Escherichia coli* production strain. Biotechnol. Bioeng., 115, 1542–1551. 29457640 10.1002/bit.26568

[btac376-B20] Vera J. et al (2003) Multicriteria optimization of biochemical systems by linear programming: application to production of ethanol by Saccharomyces cerevisiae. Biotechnol. Bioeng., 83, 335–343. 12783489 10.1002/bit.10676

[btac376-B21] von Kamp A. , Klamt S. (2017) Growth-coupled overproduction is feasible for almost all metabolites in five major production organisms. Nat. Commun., 8, 15956. 28639622 10.1038/ncomms15956 PMC5489714

